# Editorial: Single cell meets metabolism and cancer biology

**DOI:** 10.3389/fonc.2023.1125186

**Published:** 2023-02-08

**Authors:** Jieqiong Wang, Shibiao Wan

**Affiliations:** ^1^Department of Neurological Sciences, University of Nebraska Medical Center, Omaha, NE, United States; ^2^Department of Genetics, Cell Biology and Anatomy, University of Nebraska Medical Center, Omaha, NE, United States

**Keywords:** single cell analysis, metabolism, cancer biology, machine learning, bioinformatics, computational biology

Metabolism, as an essential biological process in organisms, exerts impacts on almost all physiological processes, like cellular growth and division, and the maintenance of homeostasis. Dysregulation of metabolism is highly related to various modern diseases like diabetes and various cancers. Simultaneously, cancer biology has made significant strides due to the development of a series of omics technologies (e.g., genomics, transcriptomics, and epigenomics). Heterogeneity of cancer cells within and across tumors has been recently recognized as a leading factor in resistance to therapy and disease progression. With the advent and development of single cell sequencing technologies, understanding and characterization of tumor and metabolic heterogeneity in genetic mutation, gene expression, and epigenomic levels has been achieved. Yet in-depth studies at a single cell level to understand the molecular mechanisms and facilitate clinical applications in cancer biology and metabolism remain in their infancy, largely due to the complexity and heterogeneity of the cellular phenotypes involved, which impose formidable barriers to further advancement. To fill this gap, we present this Research Topic to unravel the extent of knowledge improvement on cancer biology and metabolism based on latest development of single cell technologies.

Three papers contributed to investigating gliomas from different perspectives. Specifically, Qian et al. characterized ANXA1, a calcium-dependent phospholipid-bind protein, in >1000 Chinese gliomas patients from the molecular and clinical aspects. By leveraging both bulk and single cell RNA sequencing. (RNA-seq) technologies, Qian et al. revealed that ANXA1 highly correlates with immune-related functions and cancer hallmarks. More importantly, ANXA1 is significantly associated with clinical behaviors and patients’ survival outcomes, suggesting that it may become an important factor for gliomas diagnosis and treatment. In addition, with the help of single cell technologies, He et al. discovered an important role of SPP1-CD44-medicated crosstalk between cancer cells and macrophages in high-grade glioma. By showing the correlation between high expression levels of SPP1 and CD44 and increasing infiltration of macrophages and poor prognosis of glioma patients, He et al. identified the macrophage-mediated SPP1/CD44 signaling as a critical role of potential target in high-grade glioma treatment. In another study, Xiong et al. found that hypoxia is a poor prognosis marker for primary IDH-wild type glioblastoma (GBM) due to inducing the trend of inhibiting immune cells activity and tumor cells MES-like transformation. By using both bulk and single cell RNA-seq technologies, Xiong et al. revealed that hypoxia induced MES-like signature gene expressions in tumor cells, potentially stimulating tumor cell invasion and proliferation by cell-cell communication regulation. They also found that by upregulating immune-inhibited cell-cell interactions and inducing macrophage phenotype polarization, hypoxia inhibited immune cell activities in the tumor microenvironment.

Single cell technologies have also facilitated the progress on other cancer research and metabolism areas. For example, Fang et al. discovered a malignant cell subtype in pancreatic ductal adenocarcinoma (PDAC) with an abnormally active metabolism and enhanced glycolysis. By interrogating at the single cell resolution, Fang et al. identified five glycolytic marker genes ENO1, LDHA, PKM, PGK1, and PGM1 enriched in this highly malignant cell subpopulation. In another study, Yao et al. revealed that ferroptosis may have a promotive effect on epithelial-mesenchymal transition (EMT) in lung adenocarcinoma. Based on both bulk and single-cell RNA-seq data, Yao et al. suggested that high propensities of EMT and ferroptosis lead to non-response to immunotherapy and poor prognosis. In addition, given the close correlations between metabolism and cancer, Bai and Sun established a metabolic gene expression profiles based system for breast cancer classification. By leveraging single cell and bulk RNA-seq data, Bai and Sun further identified and verified 90 signature genes for metabolic phenotyping in breast cancer, whose impacts on immunotherapy and drug-target therapy have also been revealed. For primay liver cancer (PLC) research, Tian and Li reviewed multiple single cell sequencing based methods from different sequencing technologies for investigating the heterogeneity of cancer cells, tumor microenvironment (TME), oncogenesis, and metastatic mechanisms. They have also summarized the challenges of using single cell technologies in PLC research.

For the perspective of method development, Meng et al. proposed a pathway-based analysis method for computational drug prescription based on pan-cancer biological pathways by linking drugs to candidate pathways. They specifically identified triplets of pathways, cancers and traditional Chinese medicine to construct into a pan-cancer heterogenous network. For detecting copy number variations (CNV), Xie et al. proposed a k-nearest-neighbor based method, namely KNNCNV, to leverage next generation data and variational Gaussian mixture model for accurate CNV detection. Compared to other methods, this proposed method can accurately detect local CNVs that are masked by their surrounding regions.

It should be noted that if further wet-lab functional validations are added, contributions of the articles in this Research Topic can be significantly enhanced. However, by integrating existing single cell and bulk data in multi-omics scenarios, we believe these articles have already satisfied the goals of this Research Topic to improve our mechanistic understanding of metabolism and cancer biology based on single cell technologies. In addition, although some recent progress on single cell analysis like machine learning for processing large-scale single cells (1) has not been covered in this Research Topic, we are still excited to see the significant achievements in metabolism and cancer biology made by our contributors in this Research Topic. We are looking forward to seeing more exciting discoveries in metabolism and cancer biology by leveraging single-cell multi-omics technologies.

## Author contributions

JW and SW wrote the manuscript. SW supervised the project. All authors listed approved it for publication.
